# Energy-Aware Control of Data Compression and Sensing Rate for Wireless Rechargeable Sensor Networks

**DOI:** 10.3390/s18082609

**Published:** 2018-08-09

**Authors:** Ikjune Yoon, Dong Kun Noh

**Affiliations:** 1 Department of Smart Systems Software, Soongsil University, Seoul 06978, Korea; ijyoon@ssu.ac.kr; 2 Department of Software Convergence, Soongsil University, Seoul 06978, Korea

**Keywords:** wireless sensor networks, rechargeable, compression, sensing rate control

## Abstract

Wireless rechargeable sensor nodes can collect additional data, which leads to an increase in the precision of data analysis, when enough harvested energy is acquired. However, because such nodes increase the amount of sensory data, some nodes (especially near the sink) may blackout because more transmitted data can make relaying nodes expend more energy. In this paper, we propose an energy-aware control scheme of data compression and sensing rate to maximize the amount of data collected at the sink, while minimizing the blackout time. In this scheme, each dominant node determines the data quota that all its descendant nodes can transmit during the next period, which operates with an efficient energy allocation scheme. Then, the node receiving the quota selects an appropriate data compression algorithm and sensing rate according to both its quota and allocated energy during the next period, so as not to exhaust the energy of nodes near the sink. Experimental results verify that the proposed scheme collects more data than other schemes, while suppressing the blackout of nodes. We also found that it adapts better to changes in node density and harvesting environments.

## 1. Introduction

Wireless sensor networks (WSNs) have been used to collect environmental information in less accessible areas such as hazardous areas, battlefields, or deep water. A wireless sensor network consists of a large number of tiny wireless sensor nodes, which typically have a finite lifetime because they are battery-operated and often discarded when their batteries are exhausted. In WSN, therefore, studies have been actively carried out to extend the lifetime of nodes by reducing their energy consumption [1,2].

This has motivated the introduction of techniques for prolonging network lifetime using rechargeable nodes that obtain energy from various sources such as the sun, vibration, wind, and temperature differences [3,4,5,6]. Although the rechargeable nodes can continuously obtain energy, if they consume more energy than harvested, they can also become depleted.

To solve this problem, energy prediction [7,8,9,10] and allocation [11,12,13,14] methods have been studied, which limit the amount of available energy over time.

Kansal et al. [7] and Piorno et al. [8] introduced a harvested energy prediction scheme using a moving average approach and daily weather change, respectively. Cammarano et al. [9] proposed Pro-Energy, which can facilitate more precise prediction using long-term and short-term predictions according to weather and time. Mousavi et al. [10] proposed a new nonlinear prediction model for solar radiation on horizontal surface using ANN/SA, which is a hybrid method coupling artificial neural network (ANN) and simulated annealing (SA). Noh et al. [11] proposed a scheme to allocate the necessary amount of energy to time slots considering the historic data according to time and weather. Sharma et al. [14] provided throughput and mean delay optimal energy neutral policies for energy harvesting sensor nodes. They have been known to effectively utilize energy, irrespective of changes in the harvested energy. Some of them are especially appropriate for solar energy, which has a large variation over time because it can be only harvested in the daytime.

Another problem of WSNs is a hot-spot problem [15,16], which occurs because sensor nodes generally transmit data in a multi-hop manner; the closer nodes are to a sink node, the more data they transmit and the more energy they consume. In case of using rechargeable nodes, especially, additional data can be collected using the surplus energy harvested. If nodes far from the sink node transmit more data, nodes around the sink node should become burdened. Therefore, energy consumption of the nodes near the sink node should be considered when collecting additional data. Data compression techniques for WSNs have been studied to solve this hot spot problem [17,18]. This requires considerable processing time, and hence energy, but if energy rich nodes far from the sink compress their own data, nodes closer to the sink will use less energy in relaying that data. In contrast, the case where nodes close to the sink node compress their own data is inefficient because there leave only a few intermediate nodes to relay the compressed data. Therefore, it should be applied considering the energy efficiency of both data collection and relay nodes.

The wireless sensor nodes are implemented as a tiny embedded system, resulting in slower processing speed and less available memory. Existing compression schemes are not suitable for these low-performance devices; therefore, lightweight compression schemes are required for the wireless sensor nodes [18,19]. Sadler and Martonosi [20] introduced the sensor Lempel–Ziv–Welch (S-LZW) algorithm, which is a simplified version of the well-known dictionary-based LZW lossless compression algorithm. They also introduced S-LZW with the Burrows–Wheeler Transform (S-LZW-BWT) algorithm, which conducts invertible BWT [21] before compression by S-LZW. These are some of the most widespread compression techniques because they are designed to effectively compress sensory data. Deepu and Lian [22] presented a lossless data compression scheme for joint QRS detection aimed at wearable ECG devices. Marcelloni and Vecchio [23] introduced a compression algorithm exploiting the principles of entropy compression used for image or video compression, which uses the difference between each data. On the other hand, compression algorithms at the network level [24,25,26,27,28,29,30,31] have also been devised while the aforementioned schemes are for compressing data sensed by one node.

In this paper, we propose a data compression and sensing rate control scheme for wireless rechargeable sensor networks (WRSN) to address the above-mentioned problems and increase the precision of sensory data by increasing the amount of data acquisition. In the proposed scheme, nodes allocate the energy harvested to each time slot. Then, it determines the amount of data to be transmitted, the data compression algorithm, and the sensing period within the allocated energy. The nodes around the sink calculate and announce the transmission data quotas to their descendant nodes, and the descendant nodes collect more data than the quota, compress it to fit the quota, and transmit it. This increases the amount of data acquired in the sink without giving an overhead to the relay nodes on the transmission route.

The proposed scheme is appropriate for smart farm [32], wildfire monitoring [33,34], or structural health monitoring [35,36], where environmental data should be collected periodically.

The rest of this paper is organized as follows. In Section 2, we introduce our scheme for control of the data compression and sensing rate, and describe how a node determines the limit of data to be transmitted and its sensing rate. In Section 3, we present experimental results and assess the performance of our scheme. Section 4 concludes the paper.

## 2. Data Compression and Sensing Rate Control Scheme

**Figure 1:**
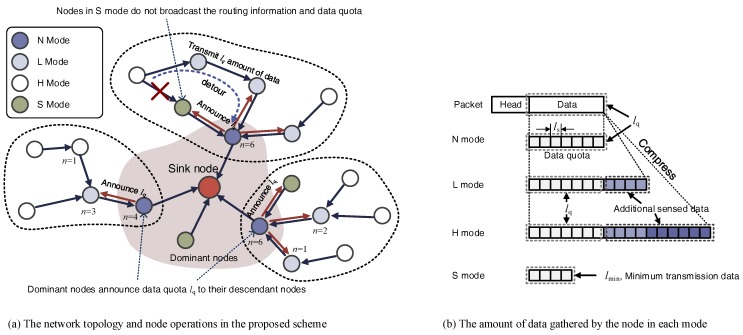
Overview of the proposed scheme.

We propose a sensing rate control and compression algorithm selection scheme to increase the amount of data gathered within the allocated energy in a WRSN application, which periodically gathers environmental information. In the proposed scheme, sensor nodes divide time into periodic time slots (e.g., by one hour) and allocate energy to the slots. Subsequently, they determine the data quota that can be transmitted within the allocated energy. To prevent the hot-spot problem, 1-hop distance node from the sink (henceforth referred to as the dominant node) limits the amount of relay data by announcing the quota to other nodes, thereby preventing the dominant node from consuming more energy than available. In addition, the nodes selectively compress and transmit data to increase the amount of sensing data within the quota. Figure 1 presents the overview of the proposed scheme.

### 2.1. Energy Consumption Model

It is assumed that rechargeable nodes have an energy buffer with capacity *c* and halt when the remaining energy $e_{r}^{}$ becomes smaller than the minimum energy $e_{\min}$. For these nodes to use energy efficiently, the amount of energy harvested should not exceed the capacity of the energy buffer. Therefore, the nodes determine how they use energy within a range where the energy does not exceed the energy buffer size and is not depleted. In this section, we determine the minimum bounds of the amount of the transmission data to prevent energy overflow.

First, the consumed energy $e_{c}^{}$ of a node during a slot can be represented as follows:

(1) $$e_{c}^{}=e_{\mathrm{tx}}^{}+e_{e},$$

where $e_{\mathrm{tx}}^{}$ is the amount of energy consumed for data transmission, and $e_{e}$ is all the energy consumed, other than that for transmission. $e_{e}$ can be obtained from the combination of the parameters (i.e., idle, sleep, and reception energy) specified in the sensor node specification and duty cycle determined in the application. $e_{e}$ becomes constant because these parameters are always steady if an application uses the same sensor nodes. $e_{\mathrm{tx}}^{}$ can be determined using the energy consumption model of Melodia et al. [37]:

(2) $$e_{\mathrm{tx}}^{}=\varphi \beta d^{\alpha },$$

where φ is the number of bytes of data to be transmitted, *d* is the transmission distance in meters, and α is the path loss exponent (2 ≤α≤ 5); the constant β (J/(bytes·m^*α*^)) is determined by the design of the node.

To obtain the minimum amount of transmission data that prevents the energy buffer from overcharging, the amount of remaining energy at the next slot should be determined. The amount of remaining energy in the battery $e_{r}^{\prime }$ is represented as follows:

(3) $$e_{r}^{\prime }=e_{r}^{}-e_{c}^{}+e_{h}^{},$$

where $e_{h}^{}$ and $e_{r}^{}$ denote the energy harvested and remaining during the current slot, respectively. If $e_{r}^{\prime }\leq c$, the remaining energy in the next slot will not exceed the energy buffer. By substituting Equations (1) and (2) into this condition,

(4) $$\varphi \geq \frac{e_{r}^{}+e_{h}^{}-c-e_{e}}{\beta d^{\alpha }}.$$

If Equation (4) is met, the remaining energy in the next slot is not overcharged. Therefore, the minimum data size $\varphi_{\min}$ can be derived as follows:

(5) $$\varphi_{\min}=\frac{e_{r}^{}+e_{h}^{}-c-e_{e}}{\beta d^{\alpha }}.$$

However, φ denotes the length that contains overhead bits such as packet headers. φ can be represented as $f_{\mathrm{packet}}\left(l\right)$ the function of the amount of sensing data *l* as follows:

(6) $$f_{\mathrm{packet}}\left(l\right)=l+\left\lceil \frac{l}{l_{\mathrm{tx}}^{\max}}\right\rceil l_{o},$$

where $l_{\mathrm{tx}}^{\max}$ is the amount of sensing data that can be sent in one packet, and $l_{o}$ is the number of overhead bits of one packet. The actual number of bits of data excluding the overhead bits $f_{\mathrm{packet}}\left(l\right)$ can be derived using Equation (6) as follows:

(7) $$f_{\mathrm{packet}}^{-1}\left(\varphi \right)=\left\{\begin{array}{cc} \varphi \left(\frac{l_{\mathrm{tx}}^{\max}}{l_{o}+l_{\mathrm{tx}}^{\max}}\right), & \mathrm{if}\;l_{\mathrm{tx}}^{\max}=Cl,C\in N\\ \left(\varphi +l_{o}\right)\left(\frac{l_{\mathrm{tx}}^{\max}}{l_{o}+l_{\mathrm{tx}}^{\max}}\right), & \mathrm{otherwise}. \end{array}\right.$$

Therefore, the minimum amount of sensing data size $l_{\min}^{}$, where remaining energy does not exceed the energy buffer capacity, is as follows:

(8) $$l_{\min}^{}=f_{\mathrm{packet}}^{-1}\left(x_{\min}\right).$$

**Figure 2:**
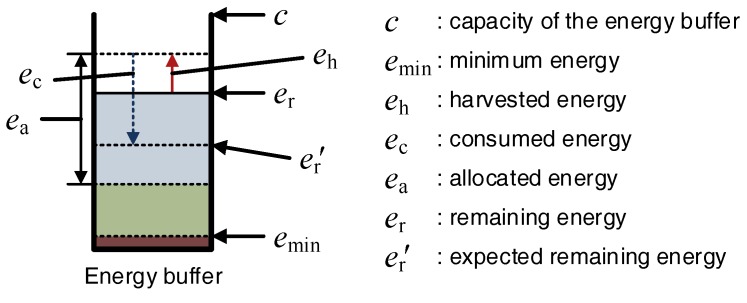
Energy model and its notations.

Figure 2 shows the energy model and its notations used in energy model.

### 2.2. Energy Allocation

**Figure 3:**
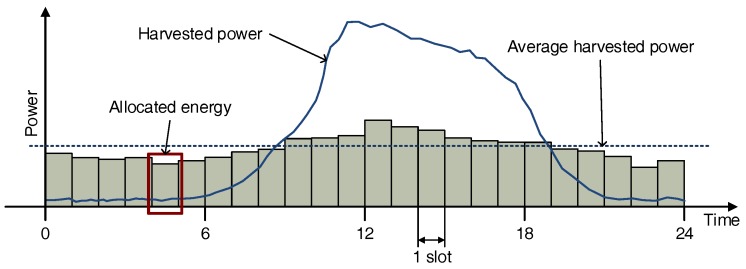
Energy allocation to the time slots.

It is possible that the energy of a node operating periodically is constantly consumed, but the energy harvested can vary over time. In particular, solar energy, which is one of the most popular renewable energy sources, dynamically varies depending on the time of day and the weather. Thus, en energy allocation scheme that determines available energy to consume during a time slot in order to achieve uniform operation independent of time is necessary. We divided a day into N slots, and sensor nodes allocate available energy $e_{a}^{}$ to each slot by using the energy allocation scheme proposed by Noh et al. [11]. Figure 3 depicts the energy allocation scheme.

For efficient use of energy, a sensor node determines the maximum amount of data $l_{\max}^{}$ that can be transmitted using only $e_{a}^{}$. Since a node must consume only the energy allocated to this slot, the condition $e_{c}^{}\leq e_{a}^{}$ must be met. By substituting Equations (1) and (2) into this condition, the following is achieved

(9) $$\varphi \leq \frac{e_{a}^{}-e_{e}}{\beta d^{\alpha }}.$$

If Equation (9) is satisfied, the node will consume energy within $e_{a}^{}$. Therefore, the maximum amount of packets to satisfy the condition $\varphi_{\max}$ can be derived as follows:

(10) $$\varphi_{\max}=\frac{e_{a}^{}-e_{e}}{\beta d^{\alpha }}.$$

The maximum amount of sensing data size excluding the overhead bits $l_{\max}^{}$ can be represented as follows:

(11) $$l_{\max}^{}=f_{\mathrm{packet}}^{-1}\left(\varphi_{\max}\right).$$

### 2.3. Node Operations

**Figure 4:**
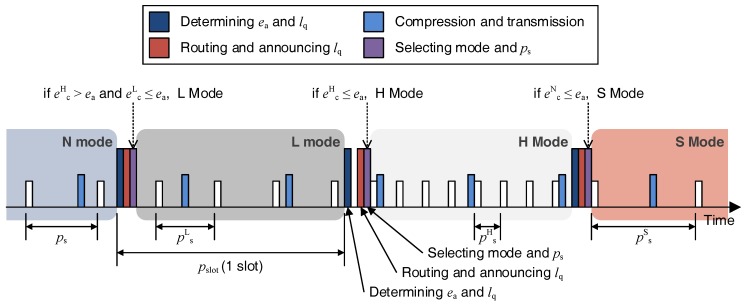
Operation of a node over time during two slots.

In the proposed scheme, each node performs the following operations every slot as shown in Figure 4.

#### 2.3.1. Energy Allocation and Determining Quota

Each node determines $e_{a}^{}$, the available energy during a slot, at the beginning of each slot, as mentioned in Section 2.2. In case of dominant nodes that can communicate directly with the sink node, they determine the amount of data that can be transmitted $l_{q}$ using $e_{a}^{}$.

#### 2.3.2. Propagating Transmission Quota

The sink node periodically broadcasts routing information, and dominant nodes receive it. Then, they relay it including $l_{q}$ to their descendant nodes. The nodes receiving it relay it to other nodes to form a minimum depth tree route.

#### 2.3.3. Mode Selection

The nodes that received $l_{q}$ determine their operating mode, compression scheme, and sensing rate, considering their energy state. There are four modes that a node can choose from, as follows:

- N mode: When the allocated energy is not enough to collect and compress additional data, the node operates in N mode of transferring gathered data without compression. Dominant nodes usually operate only in N mode because they consume more energy than other nodes and cannot save the energy of relay nodes even though they compress the data.
- L mode: If the allocated energy is sufficient to compress and transmit the data, the node operates in L mode. The node in L mode gathers additional data so that the compressed data is in size of $l_{q}$, compresses it, and transmits it. Consequently, more energy is required to collect and compress data. In this mode, nodes compress the data using the S-LZW [20] algorithm.
- H mode: In cases where it is expected that a large amount of energy will remain after compressing and transmitting the data, the node gathers more data than the L mode and compresses the data using the energy-intensive compression algorithm. As a result, it gathers more data and consumes more energy for compression than L mode. In this mode nodes compress the data using the S-LZW-BWT [20] algorithm.
- S mode: A dominant node selects S mode to save energy if the determined $l_{q}$ is less than the minimum data requirement of the application $l_{\mathrm{th}}^{}$. Other nodes select S mode to conserve energy when the allocated energy is not enough to transmit data as much as $l_{q}$. The node in S mode transmits only the smallest amount of data required by the application and is excluded from routing and does not relay data from other nodes. This can be done by not broadcasting routing messages to other nodes when they are received.

#### 2.3.4. Data Gathering and Transmission

Nodes periodically collect data, compress, and transmit the data according to the determined mode and the compression algorithm.

### 2.4. Determining Transmission Quota and Mode Selection at the Dominant Node

When a node far from the sink senses and transmits more data using extra energy, all nodes on the path from the node to the sink node consume more energy to deliver the data, resulting in a faster depletion of the energy of the nodes near the sink node. Therefore, we use a method such that dominant nodes limit the amount of traffic by determining and announcing their available transmission data amount to their descendant nodes.

Because a dominant node can transmit up to $l_{\max}^{}$ of data during a slot, as in Section 2.2, this node can send its own sensing data and data received from its descendant node up to the $l_{\max}^{}$. Therefore, if the number of descendant nodes is *n*, the amount of data that each descendant node can send $l_{q}$ is denoted as follows:

(12) $$l_{q}=\frac{l_{\max}^{}}{1+n},$$

where *n* is obtained from historic information so far. The dominant node announces $l_{q}$ to the descendant nodes during the propagating transmission quota phase so that the descendant nodes do not send data exceeding $l_{q}$.

On the other hand, if $l_{q}$ is less than the minimum amount of data required by the application $l_{\mathrm{th}}^{}$, the dominant node considers that it is insufficient to operate as a relay node and selects the S mode.

### 2.5. Mode Selection at the Normal Node

Since a node that receives $l_{q}$ can only transmit as much data as $l_{q}$ during a slot, it gathers maximum amount of additional data not exceeding $l_{q}$ and compresses it if it the allocated energy is expected to be maintained. In contrast, if it has insufficient energy to transmit $l_{q}$, it reduces energy consumption by transmitting only the minimum amount of data without relaying data from the other nodes. The node achieves this by selecting the mode mentioned in Section 2.3.

First, the node receiving $l_{q}$ decides whether to select the S mode according to whether it can transmit data of the corresponding size. If a node and its descendant nodes collect $l_{q}$ of the data, the amount of data that the node must transmit is $l_{q}\left(1+n\right)$. Therefore, the amount of energy the node consumes in this slot is as follows:

(13) $$e_{c}^{N}=f_{\mathrm{packet}}\left(l_{q}\left(1+n\right)\right)\beta d^{\alpha }+e_{e}.$$

If $e_{c}^{N}$ is greater than $e_{a}^{}$, the node *i* cannot transfer all of the data using the allocated energy. Therefore, the node chooses the S mode if the following condition is met:

(14) $$e_{c}^{N}>e_{a}^{}.$$

If a node compresses the sensing data such that the amount of compressed data is $l_{q}$ by selecting the H mode or L mode, additional energy must be consumed during the compression. If $e_{\mathrm{comp}}^{H}\left(x\right)$ and $e_{\mathrm{comp}}^{L}\left(x\right)$ denote the consumed energy for compression when compressing *x* bits data in the H mode and L mode, respectively, the consumed energy in the H mode, $e_{c}^{H}$ and the L mode, $e_{c}^{H}$ are

(15) $$e_{c}^{H}=e_{c}^{N}+e_{\mathrm{comp}}^{H}\left(l_{q}R^{H}\right)\;\mathrm{and}$$

(16) $$e_{c}^{L}=e_{c}^{N}+e_{\mathrm{comp}}^{L}\left(l_{q}R^{L}\right),$$

where $R^{H}$ and $R^{L}$ are the compression ratios in the H mode and L mode, respectively, and the compression ratio is defined as $\frac{\mathrm{Uncompressed}\;\mathrm{size}}{\mathrm{Compressed}\;\mathrm{size}}$.

If $e_{c}^{H}$ or $e_{c}^{L}$ is less than $e_{a}^{}$, it means that sufficient energy is available to operate in the corresponding mode. Therefore, the node selects the H mode if the following condition is met:

(17) $$e_{c}^{H}\leq e_{a}^{}.$$

Otherwise, if the following condition is met,

(18) $$e_{c}^{L}\leq e_{a}^{},$$

the node chooses the L mode because it means that sufficient energy is allocated to operate in the L mode, although it is insufficient to operate in the H mode. If neither of Equations (17) and (18) are satisfied, the node operates in the N mode.

### 2.6. Sensing Rate Selection

After determining the mode, the node must determine the appropriate sensing period to transmit $l_{q}$ of data in that mode. If $l_{s}$ is the amount of data sensed at once, the sensing period $p_{s}$ to collect $l_{q}$ bits during one slot is as follows:

(19) $$p_{s}=\frac{l_{s}}{l_{q}}p_{\mathrm{slot}},$$

where $p_{\mathrm{slot}}$ is the duration of one slot. A node in the N mode, which transmits data without compression, determines $p_{s}$ as its sensing period because it transmits data without compression. If a node is in the H mode or L mode, it can gather data up to $l_{q}R^{H}$ or $l_{q}R^{L}$, respectively because it compresses the data. Therefore, the sensing periods $p_{s}^{H}$, $p_{s}^{L}$ in the H mode and L mode, respectively, are derived as follows:

(20) $$p_{s}^{H}=\frac{l_{s}}{l_{q}R^{H}}p_{\mathrm{slot}}\;\mathrm{and}$$

(21) $$p_{s}^{L}=\frac{l_{s}}{l_{q}R^{L}}p_{\mathrm{slot}}.$$

A node in the S mode collects only the minimum amount of data $l_{\mathrm{th}}^{}$ to reduce the energy consumption. However, since more energy than the limit of the battery capacity cannot be stored, but the node should gather a larger amount of data compared to $l_{\min}^{}$ in Equation (8) because surplus energy cannot be stored. Thus, the sensing period in the sleep mode $p_{s}^{S}$ can be obtained by Equation (19) as follows:

(22) $$p_{s}^{S}=\frac{l_{s}}{\max(l_{\mathrm{th}}^{},l_{\min}^{})}p_{\mathrm{slot}}.$$

In this way, each node can gather the amount of data according to the requirements of the dominant node by determining the appropriate sensing period depending on its mode.

### 2.7. Pseudo-Code of the Proposed Scheme

In the proposed scheme, a node can increase the amount of gathered data within the allocated energy by transmitting only the data of the amount that does not burden the relay nodes as aforementioned in this Section. Algorithms 1 and 2 represent the entire operations of the dominant and normal nodes.
**Algorithm 1:** The operation of a dominant node 
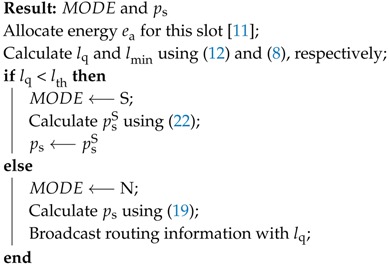

**Algorithm 2:** The operation of a normal node 
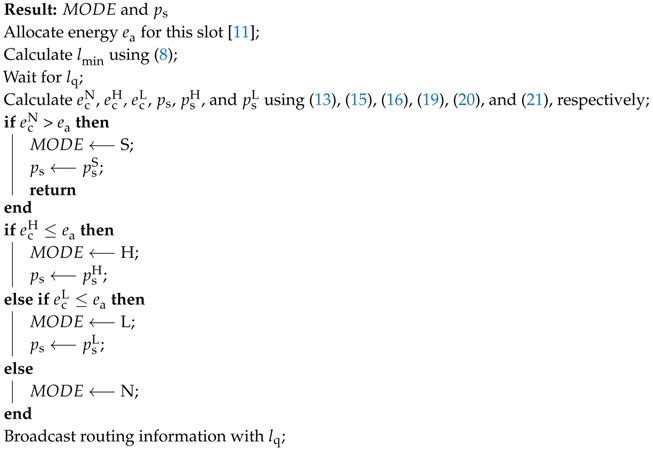



## 3. Performance Evaluation

### 3.1. Simulation Environments

We have compared the performance of our scheme with others: (1) no compression (Naive); (2) simply compressing data (S-LZW); (3) advanced compressing data (S-LZW-BWT); (4) selective compressing scheme [38] (Adaptive). We used the number of blackout nodes, the amount of data sensed at the sensor nodes, and the amount of data arriving at the sink node as measures of performance. The simulated WRSN consisted of 100 wireless rechargeable sensor nodes and one sink node, placed at random positions, and the amount of energy harvested by the nodes was modeled by the measured data [39]. Each test set ran 30 times for 2200 slots to obtain the average values. Table 1 contains the important parameters used in our simulation.

### 3.2. Simulation Results

**Figure 5:**
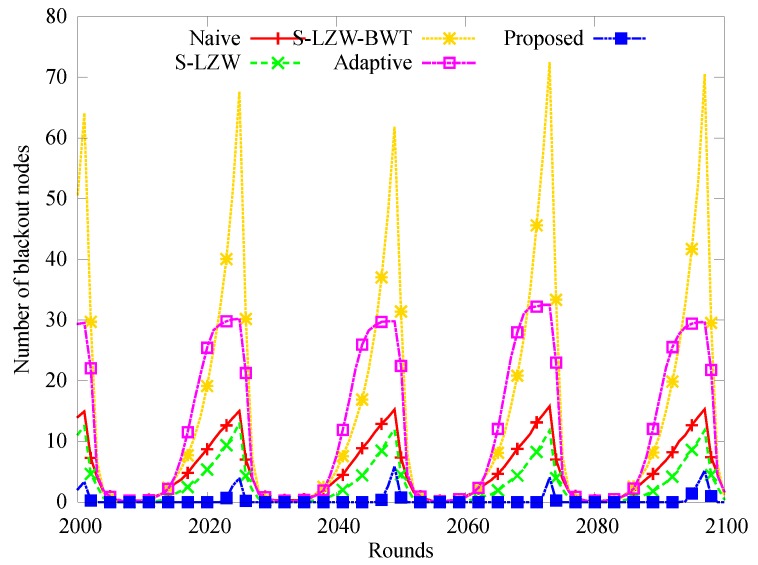
Change in the number of black-out nodes.

Figure 5 shows how the number of blackout nodes changes over time from 2000 to 2100 slots over 4 days. In other schemes except for the proposed one, the number of blackout nodes changes in a pattern similar to the change of solar energy harvested. We attribute this to the increased amount of data that had to be transferred by the nodes near the sink node. However, very few blackouts occur in the proposed scheme because the load on the nodes near the sink node is reduced by limiting the amount of data transmission, and the sleep mode is selected for the node having insufficient energy.

**Figure 6:**
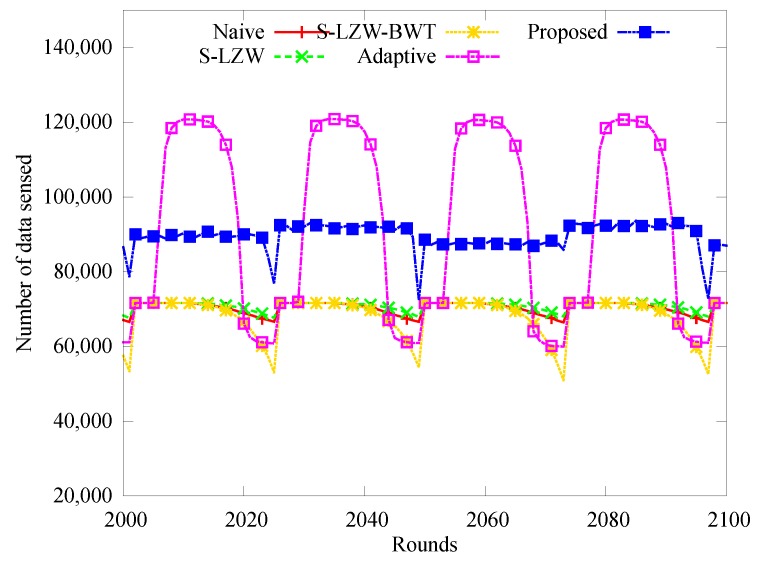
Change in the number of data sensed.

**Figure 7:**
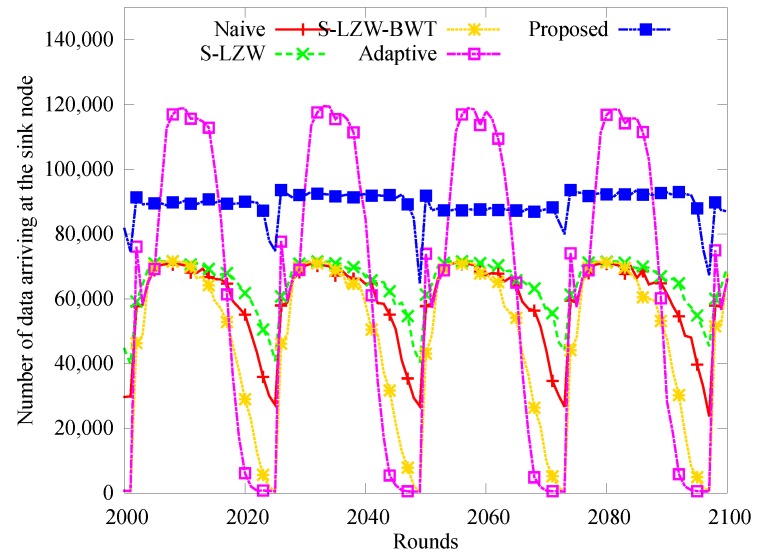
Change in the number of data arriving at the sink node.

Figure 6 and Figure 7 show how the number of sensed data and the number of data arriving at the sink node change over time. In Figure 6, the nodes of the proposed and Adaptive schemes sensed more data than those of other schemes. This is because both the schemes dynamically adjust the sensing rate according to the energy state. However, note that deviations of the data sensed over time are significant whereas the number of sensed data of the proposed scheme is almost constant. That is because Adaptive does not apply an energy allocation method. Figure 6 shows how many sensed data reach the sink node. In other schemes, many sensing data could not reach at the sink node because of the depletion of relay nodes. Conversely, most of the sensed data arrived at the sink node because very few blackouts occurred in the proposed scheme.

**Figure 8:**
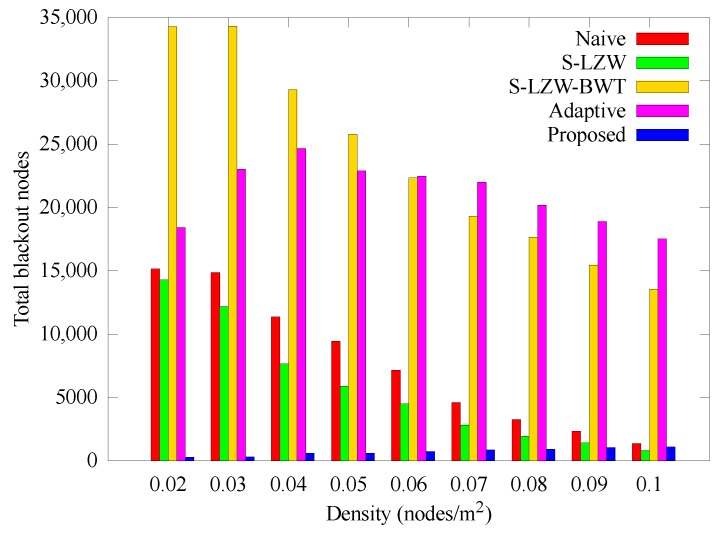
Number of cumulative blackout nodes with various node densities.

Figure 8 represents the number of blackout nodes according to the change of node density. In other schemes, it can be seen that as the density decreases, the number of blackout nodes increases. This is because if the density is low, the intermediate nodes consume more energy because the data has to go through several hops. In the proposed scheme, it can be seen that the number of blackout nodes is almost constant because the nodes adaptively allocate and use energy. However, the number of blackout nodes increases slightly as the data relayed by one node and the error of the expected transmission size increase as the density increases.

**Figure 9:**
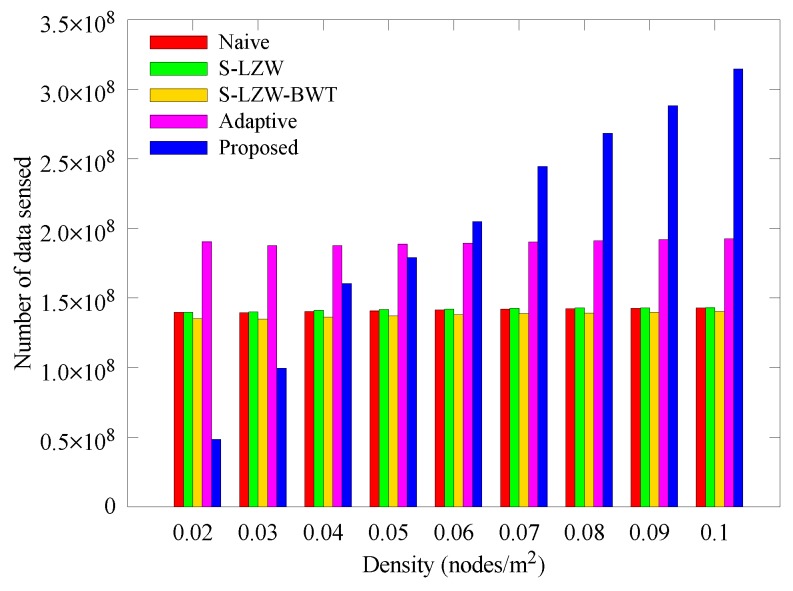
Change in the number of data sensed with various node densities.

**Figure 10:**
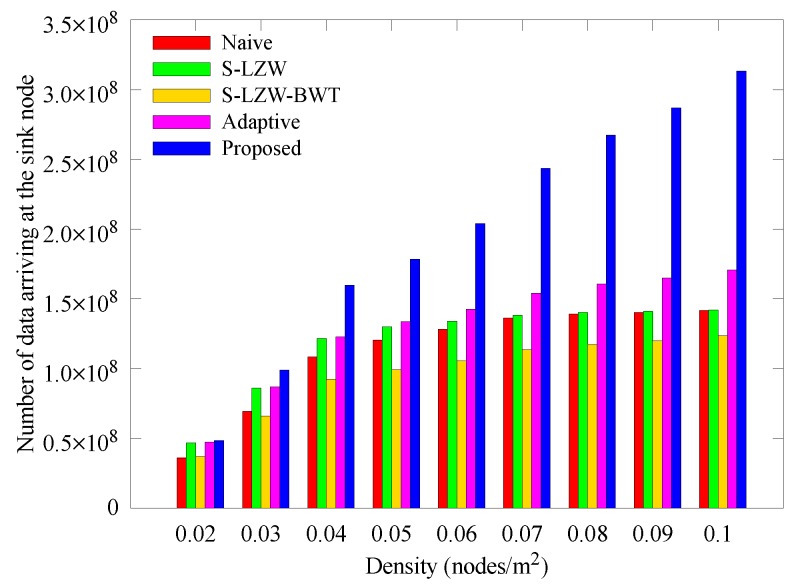
Change in the number of data arriving at the sink node with various node densities.

Figure 9 and Figure 10 respectively show how the number of sensed data and data arriving at the sink node change with nodes density. In Figure 9, the schemes except for the proposed scheme collect similar amounts of data regardless of node density. In Figure 10, however, the number of data arriving at the sink node decreases as the node density decreases. This is because, if the node density is low, the length of transmission routes get increases, more data is lost during transmission when the relay node goes to the power failure state. This is because as the node density becomes lower, the transmission route becomes longer. Therefore, when the relay node is depleted, more data is lost during transmission. However, in the proposed scheme, when the transmission route is long due to the low density, data is sensed in a small amount, and when the transmission path becomes short due to high density, a large amount of data is sensed because the amount of transmitted data is determined at dominant nodes. The result also shows that most of the sensed data arrived at the sink node because the occurrence of blackout nodes was suppressed by adjusting the amount of transmitted data. Our scheme continues to outperform the other schemes as density is increased.

**Figure 11:**
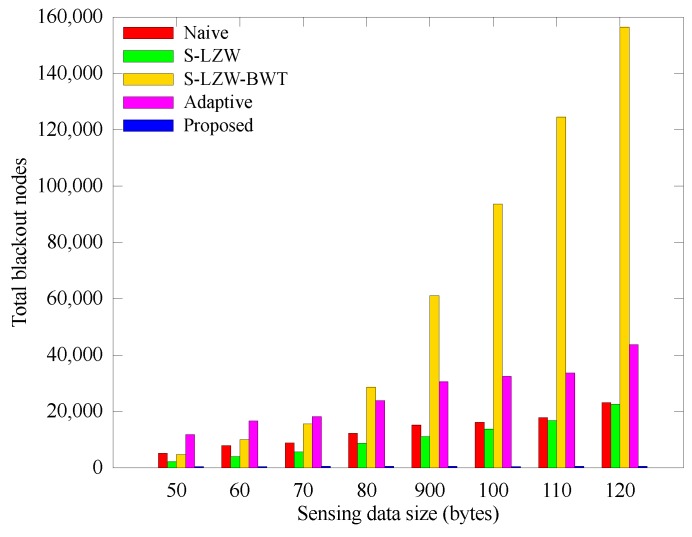
Comparison of the number of blackout nodes according to the sensing data size.

Figure 11 shows the changes in the number of blackout nodes according to the unit sensing data size. As the data size increases, the number of blackout nodes increases because the nodes must collect and transmit more data, so they consumes more energy. Nevertheless, in the proposed scheme, the nodes hardly black out independent of the unit data size due to their energy-aware operations.

**Figure 12:**
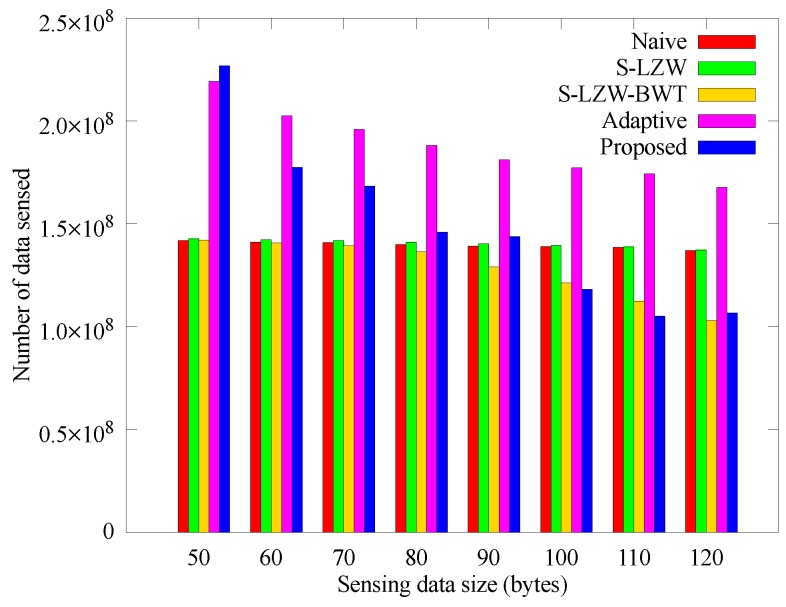
Comparison of the number of data sensed according to the sensing data size.

**Figure 13:**
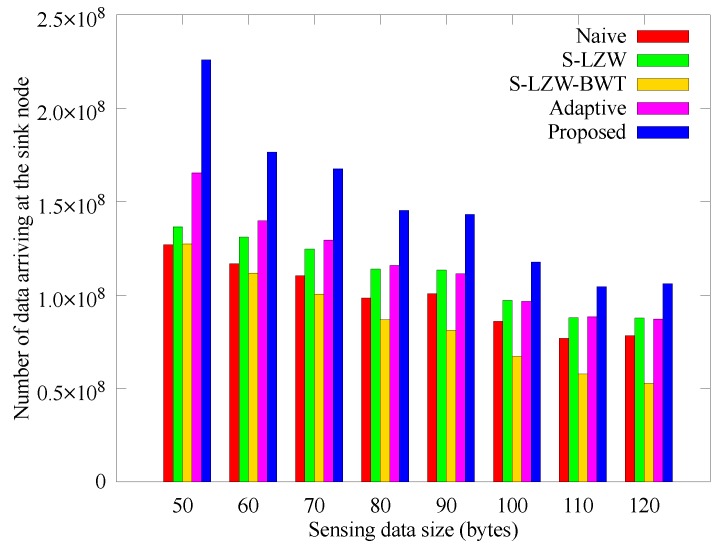
Comparison of the number of data obtained according to the sensing data size.

Figure 12 and Figure 13 respectively show how the number of sensed data and data arriving at the sink node change with the data size that the nodes sense at once. In the proposed and Adaptive schemes, larger data size leads to reduced amount of sensed data due to their adaptive operation. Even though the other schemes gather data stably, more nodes black out according to the data size as shown in Figure 11, so the amount of data obtained at the sink node is smaller than that of the proposed scheme. In the proposed scheme, however, nodes adjusts the sensing period accordingly, so the amount of data arriving at the sink node are greater than those of other schemes. Therefore, it is confirmed that the proposed scheme has high scalability for the unit data size.

**Figure 14:**
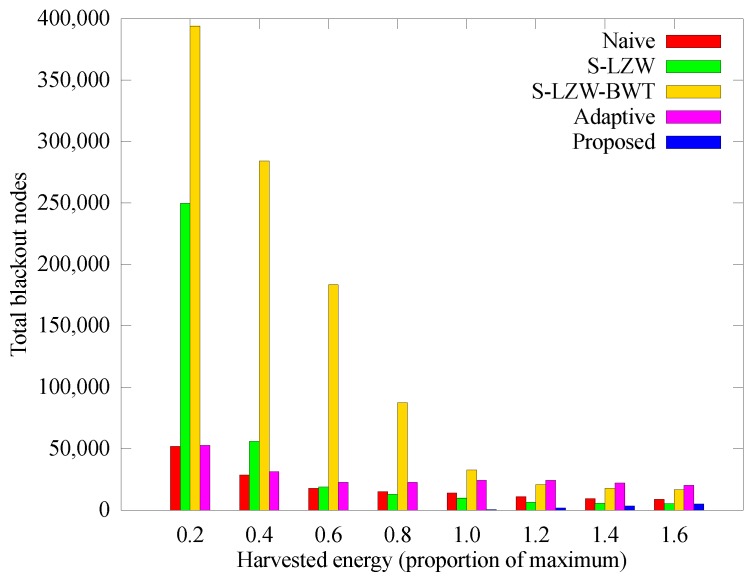
Comparison of the number of blackout nodes according to the solar energy.

Figure 14 represents the cumulative number of blackout nodes according to the change of harvested energy. The number of blackout nodes in the S-LSW and S-LZW-BWT schemes increases as the harvested energy decreases because they consume much energy in compression. However, the proposed and the Adaptive scheme adjust energy adaptively, so as to depress the number of blackout nodes independent of the harvested energy.

**Figure 15:**
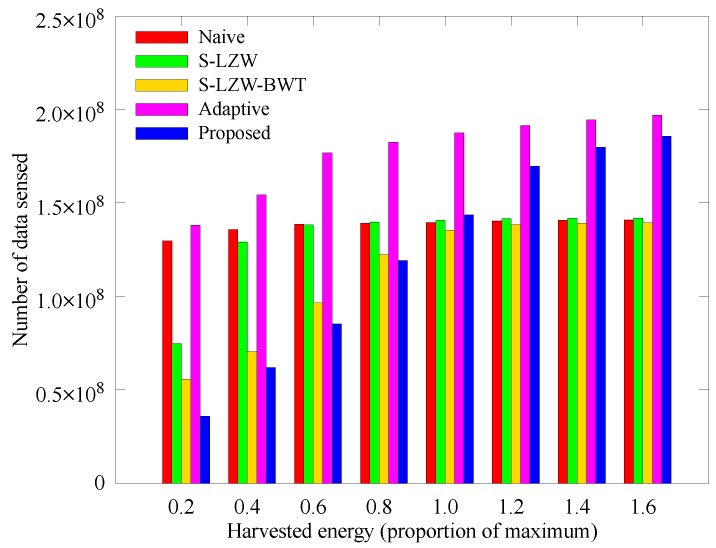
Comparison of the number of data sensed according to the solar energy.

**Figure 16:**
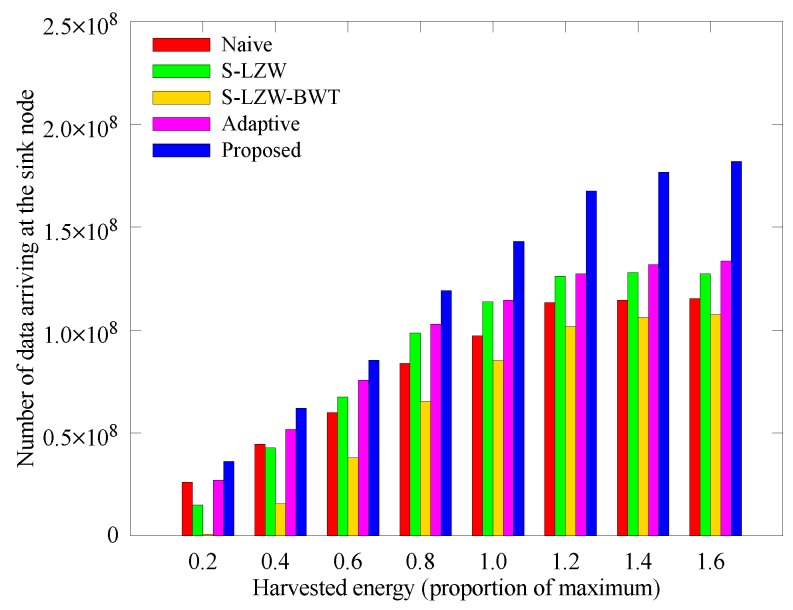
Comparison of the number of data obtained according to the solar energy.

Figure 15 and Figure 16 respectively show how the number of sensed data and data arriving at the sink node change with the amount of solar energy that the nodes can acquire. In the proposed scheme, less solar energy leads to reduced amount of sensed data because the allocated energy varies with the amount of solar energy. Although the amount of sensed data of the other schemes is larger than that of the proposed scheme, more nodes black out as shown in Figure 8, so the amount of data arriving at the sink node is smaller than that of the proposed scheme. As the solar energy increases, the allocated energy increases and the node adjusts the sensing period accordingly, so the amount of sensed data and data arriving at the sink node are greater than those of other techniques.

We have verified the performance of the proposed scheme so far. As a result, the proposed scheme shows better performance than other methods by uniformly collecting sensory data in spite of the drastic change of solar energy over time. It is inferred that this is because the appropriate operations are determined by applying harvested energy prediction and allocation methods. Even when using other energy sources (e.g., wind, temperature difference, piezo, or etc.) instead of the solar energy, the proposed scheme is expected to show good performance by applying the accurate harvested energy prediction and allocation model of the energy source.

## 4. Conclusions

We proposed a new compression and sensing rate selection scheme for WRSN. In this scheme, a node periodically selects compression algorithm and sensing rate according to its allocated and consumed energy, in order to increase the amount of data arriving at the sink node. Dominant nodes announce data quota to their descendant nodes to prevent excessive traffic. Among the nodes that have received the quota, nodes with sufficient energy gather more data than the quota, compress it and transmit it. As a result, this scheme reduces the number of nodes that blackout and thus allows more data to be obtained. However, the amount of data gathered at the dominant node drastically changes depending on the number of descendants. We plan to consider a routing that distributes the descendant nodes evenly. In addition, this scheme is designed for a flat topology and cannot be used for hierarchical topology such as clustered or layered topology. In the future, we will devise it to apply it to various structures.

## Figures and Tables

**Table 1:** Simulation parameters.

Parameters	Values
Number of nodes	200
Routing algorithm	MDT
Transmission range	10 m
Battery capacity	100 J
$p_{\mathrm{slot}}$	1 h
$l_{\mathrm{tx}}^{\max}$	102 bytes
$l_{o}$	31 bytes
$l_{s}$	80 bytes
α	4
β	8^−10^ J

## Abbreviations

The following abbreviations are used in this manuscript:

WSN	Wireless sensor Network
RWSN	Rechargeable wireless sensor network
S-LZW	Sensor LZW
S-LZW-BWT	S-LZW with the Burrows-Wheeler transform
